# The prognostic significance of pretreatment serum γ-glutamyltranspeptidase in primary liver cancer: a meta-analysis and systematic review

**DOI:** 10.1042/BSR20181058

**Published:** 2018-11-28

**Authors:** Yang Ou, Junwei Huang, Liping Yang

**Affiliations:** 1 Department of General Surgery, Wenjiang District People’s Hospital, Sichuan Province People’s Hospital of Wenjiang Branch, Chendu, China; 2 Department of General Medicine, Wenjiang District People’s Hospital, Sichuan Province People’s Hospital of Wenjiang Branch, Wenjiang District 611130, Chendu, Sichuan Province, China

**Keywords:** Gamma-glutamyltranspeptidase, Hepatocellular carcinoma, Intrahepatic cholangiocarcinoma, prognosis

## Abstract

**Aim:** To assess the prognostic value of the pretreatment serum γ-glutamyltranspeptidase (GGT) level in patients with primary liver cancer (PLC). **Methods:** Relevant studies were systematically searched online on Web of Science, PubMed, and Embase databases published until 9 October 2018. The end points were overall survival (OS), recurrence-free survival (RFS), and disease-free survival (DFS). Meta-analysis was conducted using hazard ratio (HR), and its 95% confidence interval (CI) as effect measure. **Results:** A total of 33 eligible studies with 9238 patients with PLC were included in this meta-analysis. The synthesized analysis showed that that higher serum GGT level was significantly related to poorer OS (HR: 1.79, 95% CI: 1.66–1.93, *P*<0.01), RFS (HR: 1.60, 95% CI: 1.46–1.77, *P*<0.01), and DFS (HR: 1.52, 95% CI: 1.33–1.73, *P*<0.01) of patients with PLC. Subgroup analyses demonstrated that the negative prognostic impact of higher serum GGT level on OS and RFS was still of significance regardless of ethnicity, pathological type, sample size, cut-off value, first-line treatment, and analysis type. **Conclusion:** The pretreatment serum GGT might be a predictive factor of poor prognosis for PLC patients.

## Introduction

Primary liver cancer (PLC), including hepatocellular carcinoma (HCC) and intrahepatic cholangiocarcinoma (ICC), is one of the most common human malignant neoplasms [1]. Although a comprehensive therapy integrating surgical resection, thermal ablative techniques, chemotherapy, and molecular-targetted therapy has been applied to deal with patients with PLC in recent years, the long-term survival of patients remains rather unfavorable [2,3]. Hence, it is of great importance to identify biomarkers for accurately predicting the prognosis of patients with PLC, which may contribute to optimizing individual treatment and then improve the long-term outcomes.

γ-Glutamyltranspetidase (GGT) is a cell-membrane bound enzyme that modulates the metabolism of glutathione (GSH), catalyzes the degradation of extracellular GSH, and subsequently facilitates amino-acid recovery for intracellular GSH synthesis [4]. GGT has been recognized to enhance cellular antioxidant defenses [4]. In addition, several researchers reported that GGT might be involved in contributing to the tumor initiation, progression, invasion, and drug resistance [5–7]. More importantly, increased serum level of GGT was found to be linked with worse prognosis in several human malignancies, including PLC.

Up to date, numerous studies have reported that serum GGT level was related to the prognosis of patients with PLC [8–38], while the limitation of sample size in individual studies might affect the reliability of the relevant conclusions due to weak statistical power. Therefore, to conquer the potential effect of sample size, we conducted a meta-analysis to further investigate the prognostic value of serum GGT in PLC.

## Materials and methods

### Search strategy

We searched for relevant literature in PubMed, Embase, and Web of Science from inception to 9 October 2018. The detailed search strategy was presented in Supplementary Material. Only publications written in English were considered.

### Inclusion and exclusion criteria

Articles meeting the following criteria were defined as eligible ones for this meta-analysis: (i) cohort study or observational study; (ii) tumors were confirmed as HCC or ICC through histology; (iii) studies reported the relationship between serum GGT level and prognosis of PLC patients; and (iv) studies provided hazard ratio (HR) estimation with 95% confidence interval (CI) of OS, disease-free survival (DFS), or recurrence-free survival (RFS).

The exclusion criteria included: (i) duplicated publications from different databases; (ii) articles unpublished or published in non-English, conference abstracts, and case reports; (iii) animal or cell experiments; (iv) when several studies enrolled the same or overlapping patients, only the latest or most complete ones were selected; (v) studies only providing HR estimation with 95% CI based on variate analysis. Two reviewers searched for relevant studies independently and disagreements were worked out through discussion.

### Data extraction

Two reviewers extracted the required data from all eligible studies independently and inconsistencies were worked out by discussion. The required information included first author’s family name, publication time, country, recruitment time, median age, sample size, disease stage, primary treatment type, GGT cut-off level, follow-up time, and HR estimations for prognostic indicators, including overall survival (OS), DFS, and RFS.

### Quality assessment

The methodological quality of all eligible studies was evaluated by two reviewers using Newcastle–Ottawa scale (NOS) independently [39]. The maximum of 9 stars was applied to assess the selection, comparability as well as exposure, and outcome of each included study. In this meta-analysis, we defined studies with no less than 7 stars as high quality and 6 stars as moderate quality.

### Statistical analysis

We measured the effects of serum GGT level in OS, DFS, and RFS using the pooled HRs and 95% CIs. Heterogeneity was evaluated with *I^2^* test. The random effect model was chosen for pooling analysis if significant heterogeneity existed (*I^2^* > 50% or *P-*value of heterogeneity test <0.05). If not (*I*^2^ ≤ 50% or *P*-value of heterogeneity test), the fixed effect model was applied to perform pooling analysis. When the pooled HRs and 95% CIs were >1, it indicated that PLC patients with higher level of serum GGT had poorer prognosis as compared with those with lower level. Publication bias was evaluated using Begg’s funnel plot and Egger’s test [40,41]. When there is significant publication bias, trim-and-fill method was utilized to evaluate the effect of publication bias on the robustness of the pooled HR [42]. In addition, subgroup analysis and sensitivity analysis were also performed to assess the influence of each study on the pooled HR. *P* <0 .05 was considered as statistically significant. The subgroup analysis and sensitivity analysis in this meta-analysis were fulfilled by means of STATA version 12.0 (Stata Corporation, College Station, TX), and the other statistical analysis were carried out with Review Manager (RevMan) version 5.3 (The Nordic Cochrane Centre, The Cochrane Collaboration, Copenhagen).

## Results

### Search results

**Figure 1:**
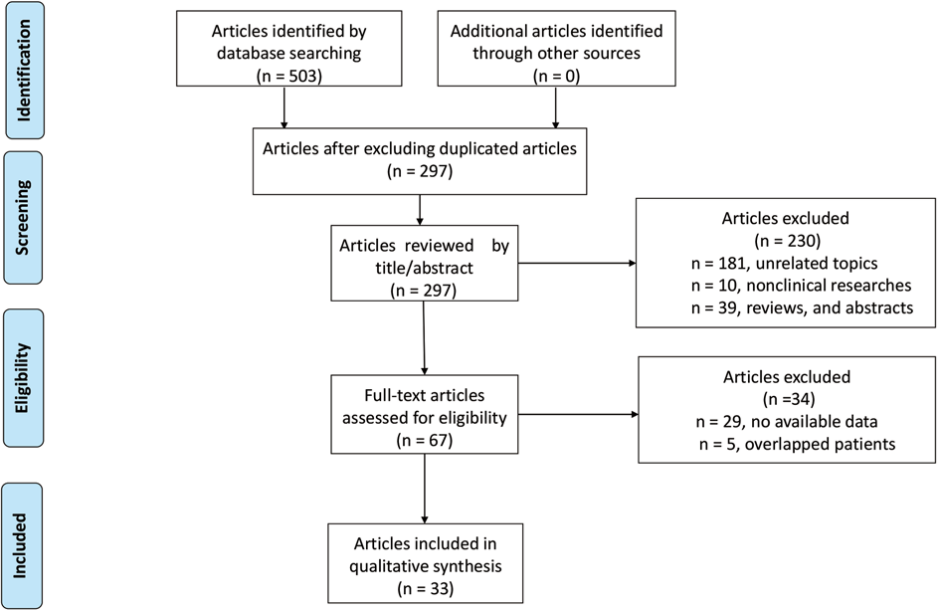
Flow diagram of literature search process

The processes of searching and filtering of publications were presented in Figure 1. Initially, a total of 503 articles were identified. Then we used Endnote X7 software to exclude 217 duplicated publications with 297 articles left for further identification. Subsequently, after scanning the titles, abstracts, and publication types of these 297 articles, 230 articles were excluded for meeting abstracts, reviews, case reports, or comments (*n*=39), irrelevant topics (*n*=181), and cell and animal experiments (*n*=10), and 60 articles remained for full-text review. In the process of full-text review, 34 studies were further excluded for no available data (*n*=29) and enrolling the same or overlapping population (*n*=5). Ultimately, a total of 33 eligible studies with 9238 patients were included in this meta-analysis [8–38,43,44].

### Study characteristics and quality assessment

The detailed characteristics of the 33 eligible studies were shown in Table 1. These studies were originally published between 2011 and 2018. Of these studies, 30 studies with 8056 patients were from China, and 3 studies with 1182 patients from Italy, France, and U.S.A. A total of 28 studies reported about HCC and 5 studies focussed on ICC. In addition, OS was mentioned in 29 studies, RFS was found in 13 articles, and DFS was reported in 5 studies. We assessed the quality of the 33 included studies in our meta-analysis following the guideline of the NOS. The included studies were given 6–8 scores, indicating that the eligible studies were of moderate to high quality.

**Table 1:** The main characteristics of the included studies

Study	Country	Median age	Number of patients	Tumor type	Disease stage	Primary treatment	Cut off value (U/l)	Follow up (month)	Survival outcomes	Variables adjusted in multivariate analysis	NOS
Carr et al. (2013) [8]	Italy	NR	344	HCC	Unresectable	TACE	150	NR	OS*	Sex, age, alcohol, smoking	6
Chen et al. (2014) [9]	China	55	154	HCC	Unresectable	TACE	85	NR	OS*	ALT, ALB, gross tumor volume, AFP	6
Dong et al. (2017) [10]	China	NR	654	HCC	Early stages (BCLC)	Liver resection	50	NR	OS*, DFS*	Sex, ABS, and liver cirrhosis	8
Dvorchik et al. (2007) [11]	U.S.A.	NR	750	HCC	Unresectable	TACE	100	NR	OS*	AFP, ascites, liver cirrhosis	8
Fan et al. (2017) [12]	China	52	161	HCC	Small HCC	Liver resection followed by TCEA	60	36.6	OS	Liver cirrhosis, and recurrence	7
Fu et al. (2016) [13]	China	51	308	HCC	TNM I-IV	Liver resection	88	29	OS*, DFS*	Child-Pugh stage, tumor number, tumor size, and AFP	7
Fu et al. (2016) [14]	China	49.5	130	HCC	Milan criteria (within and beyond)	Liver transplantation	128	40.3	OS*, DFS*	Tumor size, AFP	6
Gan et al. (2018) [44]	China	NR	326	HCC	BCLC A-C	Liver resection	45	48	RFS*	Liver cirrhosis, GGT, tumor size, and microvascular invasion	8
Guiu et al. (2012) [15]	France	68.2	88	HCC	NR	TACE	165	11.66	OS	Age, WHO PS, tumor burden, AFP, tumor number, and tumor size	6
He et al. (2013) [16]	China	NR	127	HCC	BCLC A-C	Liver resection	50	NR	OS, RFS	Tumor number, tumor size, tumor differentiation and vascular invasion	6
Hu et al. (2017) [17]	China	60	422	ICC	NR	Liver resection	50	NR	OS*, RFS*	Tumor number, tumor size, CA19-9, CEA	7
Ju et al. (2009) [18]	China	NR	219	HCC	BCLC A-C	Liver resection	60	26.76	OS*	Hepatitis B antigen, tumor differentiation, BCLC stage, GGT/ALT ratio	8
Li et al. (2014) [19]	China	55	283	ICC	TNM I–IV	Liver resection	50	17	OS*, RFS	Tumor number, LNM, vascular invasion, adjuvant TACE	8
Liu et al. (2013) [21]	China	59	81	ICC	NR	Liver resection	64	12.2	OS		6
Liu et al. (2012) [20]	China	50.79	338	HCC	NR	Liver resection	80	51	OS		8
Ma et al. (2014) [22]	China	NR	254	HCC	NR	RFA	75	27	OS*, RFS*	TB, tumor size and albumin ALT	7
Shi et al. (2017) [23]	China	60	271	HCC	TNM I–III	Liver resection	50	26	OS*	Tumor encapsulation, tumor number, tumor size, vascular invasion, TNM stage, ALC, AMC, LMR, ALT, and AST	7
Song et al. (2015) [24]	China	65	384	HCC	TNM I–III	Liver resection	100	57.5	OS*, RFS*	CA 19-9, microvascular invasion, ICG-R15, and intrahepatic metastasis	8
Su et al. (2013) [25]	China	56	333	HCC	TNM I–III	Liver resection	60	45.9	RFS*	ICG-15R, anti-viral therapy, macroscopic venous invasion, and microscopic venous invasion	8
Tian et al. (2017) [26]	China	NR	189	HCC	BCLC A-C	Liver resection	54	30.9	RFS*	High-density lipoprotein	7
Wang et al. (2012) [28]	China	53	441	HCC	BCLC A-C	TACE	75	12	0S*	AFP and tumor size	7
Wang et al. (2016) [27]	China	NR	221	HCC	BCLC A-C	WMA	50	41	OS*, RFS*	AFP, tumor size, tumor number, ALP, Ablation effectiveness and recurrence types	7
Wu et al. (2016) [29]	China	NR	469	HCC	BCLC A-C	Liver resection	81.5	42	OS*, RFS*	Tumor size, tumor number, vascular invasion, ALB, AST, ALT, ALP, LDH and AFP	8
Xu et al. (2014) [30]	China	53.5	172	HCC	NR	Liver resection	117	34.92	OS*	HBsAg, ALP, and TS	7
Yin et al. (2013) [31]	China	56	411	ICC	TNM I–III	Liver resection, palliative chemotherapy, TACE, supportive care	50	26	OS*, RFS*	Pathological subtype, TNM stage, tumor differentiation, and vascular invasion	6
Zhang et al. (2014) [33]	China	56.8	138	HCC	NR	TACE	50	12	OS*	PVTT, tumor size, tumor number and diabetes mellitus, NLR	6
Zhang et al. (2016) [35]	China	53	601	HCC	TNM I–IV	Liver resection	50	NR	DFS*	Gender, smoking, AFP, cirrhosis, tumor size, PVTT, microvascular tumor thrombus, TNM stage	8
Zhang et al. (2017) [32]	China	58.83	173	ICC	Unresectable	Chemotherapy	113	NR	OS*	Alb, ALP, TB, DB, chemotherapy	7
Zhang et al. (2011) [34]	China	54	277	HCC	BCLC B	TACE	50	18.7	OS*	Ascites, albumin, TS, AFP	7
Zhang et al. (2015) [36]	China	51	38	HCC	TNM I–III	Liver resection followed by adjuvant sorafenib therapy	40	28.6	OS*, RFS*	PVTT, tumor number, liver cirrhosis, Increased NLR after Sorafenib, and increased GGT after sorafenib	6
Zhong et al. (2018) [37]	China	NR	175	HCC	BCLC A–C	Liver resection	60	NR	OS, DFS*	AFP, CA-199, tumor size, tumor encapsulation, HBsAg, PVTT	7
Zhou et al. (2012) [38]	China	53	114	HCC	TNM I–III	Liver resection	50	NR	OS	Tumor size, PVTT, and liver cirrhosis	6
Zhou et al. (2018) [43]	China	NR	182	HCC	TNM I–IV	Liver resection	54	NR	OS*, RFS*	AFP and tumor size	7

Abbreviations: ABS, albumin-bilirubin score; AFP, α–fetoprotein; ALB, albumin; ALC, absolute lymphocyte count; ALP, alkaline phosphatase; AMC, absolute monocyte count; BCLC, Barcelona clinic liver cancer stage; CA19-9, carbohydrate antigen 19-9; CEA, carcinoembryonic antigen; DB, direct bilirubin; ICG-R15, indocyanine green retention rate at 15 min; LMR, lymphocyte-to-monocyte ratio; LNM, lymph node metastasis; NLR, neutrophil to lymphocyte ratio; NR, not reported; PVTT, portal vein tumor thrombus; RFA, radiofrequency ablation; TACE, transcatheter arterial chemoembolization; TB, total bilirubin; TNM stage, tumor node metastasis stage; WHO PS, World Health Organization Performance Status; WMA, microwave ablation. *, multivariate analysis.

### Meta-analysis

#### Relationship between serum GGT and OS in PLC patients

**Figure 2:**
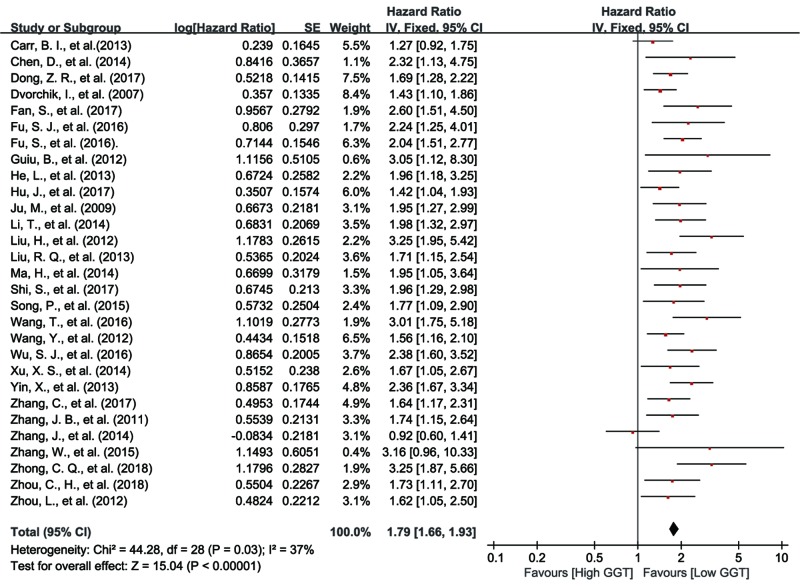
Forest plot of the HR for the relationship between pretreatment serum GGT level and OS in patients with PLC

There were a total of 29 studies assessing the relationship between OS and serum GGT level [8–24,27–34,36–38,43]. The heterogeneity of these 29 studies was significant (*I^2^ * =  37%, *P*=0.03), so the random effect model was applied. The pooled HR was 1.79 (95% CI: 1.66–1.93, *P*<0.01), indicating that higher serum GGT level was significantly related to worse OS in PLC patients (Figure 2).

#### Relationship between serum GGT and RFS in PLC patients

**Figure 3:**
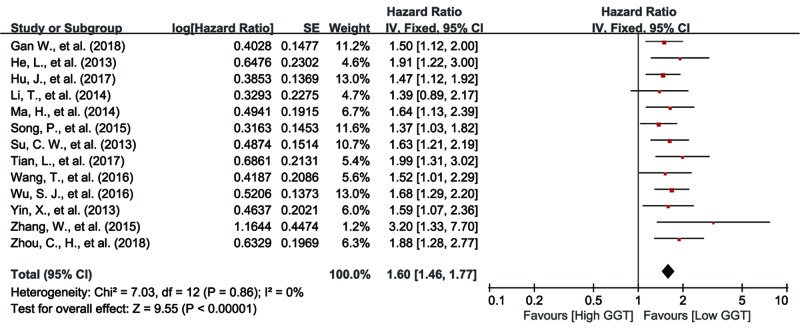
Forest plot of the HR for the relationship between pretreatment serum GGT level and RFS in PLC patients

A total of 13 eligible studies referred to the association between serum GGT level and RFS [16,17,19,22,24–27,29,31,36,44]. Because no significant heterogeneity amongst these 13 studies was observed (I^2^  =  0%, *P*=0.86), we used the fixed effect model to conduct the pooling analysis. The result showed that PLC patients with higher GGT level had more unfavorable RFS (HR: 1.60, 95% CI: 1.46–1.77, *P*<0.01) (Figure 3).

#### Relationship between serum GGT and DFS in PLC patients

**Figure 4:**
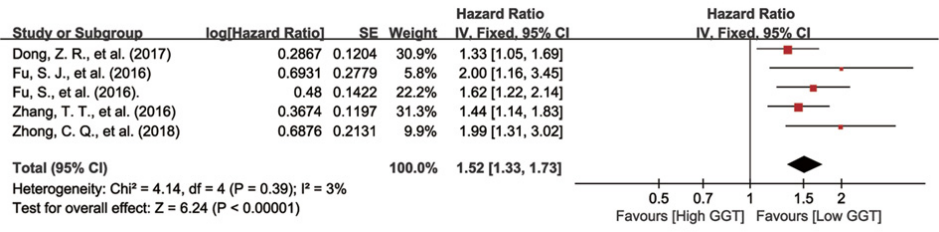
Forest plot of the HR for the relationship between pretreatment serum GGT level and DFS in PLC patients

The association between serum GGT level and DFS was investigated in five of the included studies [10,13,14,35,37]. Considering that there was no obvious heterogeneity amongst these five studies (I^2^  =  3%, *P*=0.39), we applied fixed effect model to pool the data. From the result, it was found that there was correlation between serum GGT level and DFS in PLC patients (HR: 1.52, 95% CI: 1.33–1.73, *P*<0.01) (Figure 4).

### Subgroup analysis

In order to explore the potential sources of heterogeneity of the combined HR for OS, we conducted subgroup analyses through stratifying eligible studies by ethnicity (Asian and non-Asian), pathological type (HCC and ICC), sample size (≤300 and >300), cut-off value (≤50, 50–75, 70–100, and >100), disease stage (only unresectable), primary treatment (Liver resection, transcatheter arterial chemoembolization (TACE), and ablation), and analysis type (multivariate analysis and univariate). It should be noted that the subgroup of disease stage (unresectable) only covered four included studies that referred to OS in patients with unresectable HCC. Each of the remaining 25 eligible studies referring to OS enrolled patients with different disease stages, or even did not provide clear information about disease stages, so these studies showed no similarity in terms of disease stage and we could not classify these studies into subgroup of disease stage for pooling analysis. The same situation is also for subgroup of primary treatment. We established this subgroup according to TACE, liver resection, and ablation, which only covered 26 eligible studies that referred to OS. However, the remaining three eligible studies that referred to OS showed no similarity in primary treatment, so we could not classify these studies into subgroup of primary treatment for pooling analysis either. From the results, we found that there was no subgroups in which the heterogeneity of the combined HR for OS (Table 2) disappeared completely, indicating that those factors might not be the sources of heterogeneity of the pooled HR for OS. Although we failed to identify the possible sources of the pooled HR for OS, we demonstrated that our pooled result was robust, since the pooled HR for OS was >1 continuously, and its correspondent CI did not overlap 1 in all subgroups (Table 2).

**Table 2:** The association between GGT and OS in different subgroups

Analysis	Number of studies	HR (95% CI)	Test of null (two-tail)		Heterogeneity			Model
			Z-value	*P*-value	I^2^ (%)	*P*-value	df	
**(i) Ethnicity**								
Asian	26	1.89 (1.71, 2.09)	12.49	<0.01	29	0.09	25	Fixed
Non-Asian	3	1.43 (1.11, 1.84)	2.74	<0.01	26	0.26	2	Fixed
**(ii) Pathological type**								
HCC	24	1.81 (1.62, 2.01)	10.70	<0.01	32	0.07	23	Fixed
ICC	5	1.97 (1.51, 2.56)	5.05	<0.01	60	0.04	4	Random
**(iii) Sample size**								
>300	10	1.77 (1.50, 2.09)	6.72	<0.01	54	0.02	9	Random
≤300	19	1.89 (1.67, 2.14)	10.22	<0.01	22	0.19	18	Fixed
**(iv) Cut-off value**								
≤50	12	1.86 (1.55, 2.23)	6.66	<0.01	54	0.01	11	Random
50–75	6	1.79 (1.50, 2.12)	6.64	<0.01	0	0.71	5	Fixed
75–100	6	2.03 (1.59, 2.59)	5.73	<0.01	53	0.06	5	Fixed
>100	5	1.62 (1.30, 2.03)	4.25	<0.01	18	0.30	4	Fixed
**(v) Analysis type**								
Univariate	7	2.25 (1.78, 2.85)	6.75	<0.01	30	0.20	6	Fixed
Multivariate	22	1.75 (1.58, 1.94)	10.64	<0.01	31	0.08	21	Fixed
**(vi) Primary treatment**								
TACE	7	1.58 (1.14, 2.20)	8.93	<0.01	55	0.06	4	Random
Liver resection	17	1.92 (1.67, 2.22)	9.13	<0.01	0	0.88	6	Fixed
Ablation	2	3.13 (2.16, 4.55)	6.00	<0.01	0	0.84	1	Fixed
**(vii) Disease stage**								
Unresectable	4	1.47 (1.24, 1.74)	4.45	<0.01	0	0.43	3	Fixed

Abbreviation: df, degree of freedom.

Although no significant heterogeneity was observed for the pooled HR for RFS, we still performed subgroup analysis to test whether our pooled HR for RFS was stable and dependable. The results showed that significant heterogeneity was still not detected and the pooled HR for RFS was >1 continuously with its corresponding CI not overlapping 1 in any subgroup (Table 3), suggesting that our pooled result was reliable.

**Table 3:** The association between GGT and RFS in different subgroups

Analysis	Number of studies	HR (95% CI)	Test of null (two-tail)		Heterogeneity			Model
			Z-value	*P*-value	I^2^ (%)	*P*-value	df	
**(i) Pathological type**								
HCC	10	1.64 (1.47, 1.84)	8.77	<0.01	0	0.74	9	Fixed
ICC	3	1.48 (1.22, 1.81)	3.88	<0.01	0	0.9	2	Fixed
**(ii) Sample size**								
>300	6	1.53 (1.36, 1.73)	6.98	<0.01	0	0.93	5	Fixed
≤300	7	1.75 (1.48, 2.06)	6.64	<0.01	0	0.67	6	Fixed
**(iii) Cut-off value**								
≤50	7	1.56 (1.35, 1.80)	6.13	<0.01	0	0.69	6	Fixed
50–75	4	1.75 (1.46, 2.09)	6.07	<0.01	0	0.84	3	Fixed
75–100	2	1.53 (1.26, 1.86)	4.25	<0.01	4	0.31	1	Fixed
**(iv) Analysis type**								
Univariate	2	1.63 (1.18, 2.23)	3.01	<0.01	0	0.33	1	Fixed
Multivariate	11	1.60 (1.45, 1.77)	9.07	<0.01	0	0.81	10	Fixed
**(v) Primary treatment**								
Liver resection	10	1.61 (1.44, 1.79)	8.68	<0.01	0	0.64	9	Fixed
Ablation	2	1.58 (1.20, 2.09)	3.26	0.01	0	0.79	1	Fixed

Abbreviation: df, degree of freedom.

For limited number of the eligible studies about DFS, subgroup analysis was not conducted to investigate the sources of the pooled HR for DFS.

### Sensitivity analysis

**Figure 5:**
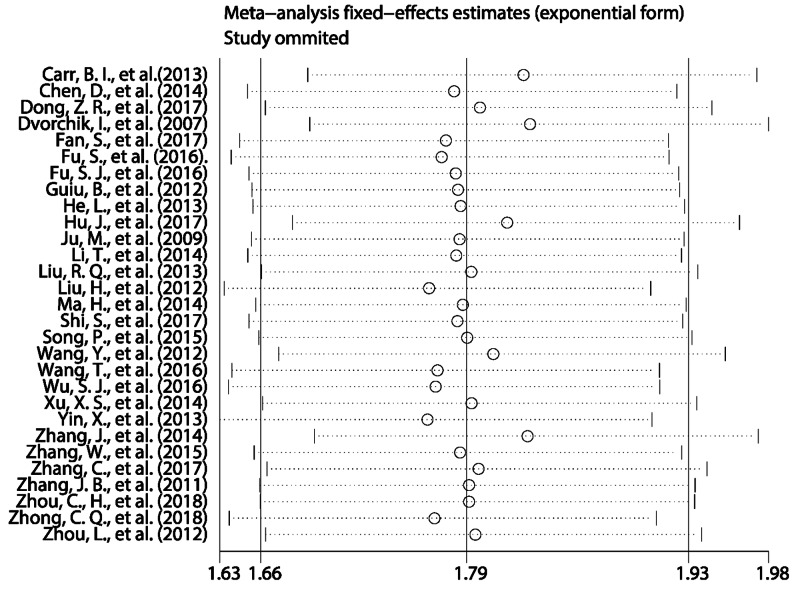
Sensitivity analyses to assess the effect of individual studies on the overall pooled HR for OS in PLC patients

**Figure 6:**
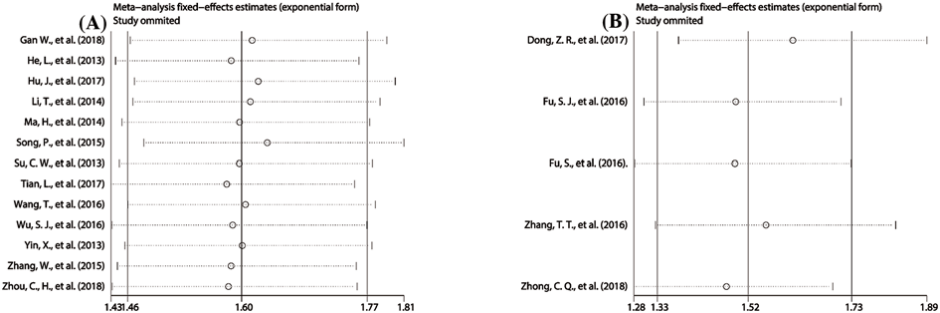
Sensitivity analyses to assess the effect of individual studies on the overall pooled HR for DFS (**A**) and RFS (**B**) in PLC patients

Sensitivity analysis was used to evaluate the robustness of the pooled results. The results showed that the pooled HRs for OS (Figure 5), RFS (Figure 6A), and DFS (Figure 6B) did not alter substantially when the included studies were sequentially omitted in each step, revealing the robust stability of our pooled results.

### Publication bias

**Figure 7:**
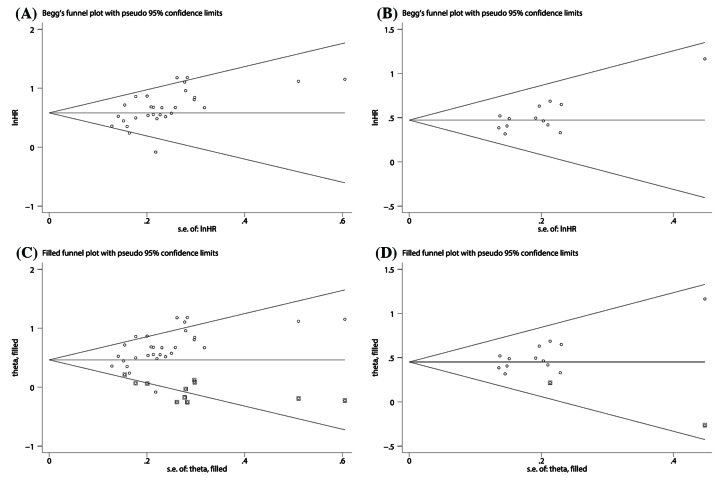
The publication bias assessment of the pooled HRs for OS (A) and RFS (B)and the trim-and-fill analysis of the effect of publication bias on the pooled HRs for OS (C) and RFS (D)

The assessment for publication bias was fulfilled by Begg’s funnel plot and the Egger’s tests. As our results show, there were obvious asymmetries for Begg’s funnel plots of OS (Figure 7A) and RFS (Figure 7B). Additionally, the Egger’s test also suggested that there was significant publication bias for OS (*P*<0.01) and RFS (*P*<0.01) in this meta-analysis. Therefore, we applied trim-and-fill method to assess the impacts of the publication bias on the reliability of the pooled HR for OS and RFS. From the results, we observed that the adjusted funnel plots for OS and RFS turned symmetric (Figure 7C,D). Furthermore, the result of trim-and-fill method showed that the adjusted pooled HRs for OS and RFS were still >1, and meanwhile their corresponding CIs did not include 1. From the results stated above, we concluded that the publication bias did not substantially affect the robustness of the pooled HR for OS and RFS. Considering the limited number of the included studies about DFS, we did not perform the Begg’s test and Egger’s test to assess the publication bias for DFS.

## Discussion

To our best knowledge, the present study is the first meta-analysis to synthetically analyze the prognostic significance of serum GGT in patients with PLC. The results of this meta-analysis demonstrated that a higher serum GGT is a useful biomarker for poorer OS, RFS, and DFS in PLC patients and this result would not change substantially when any eligible study was sequentially omitted in sensitivity analysis. Moreover, our subgroup analyses validated that higher serum GGT remained an effective prognostic indicator in spite of ethnic background, sample size, pathological type, cut-off value, primary treatment, and analysis type.

Several potential mechanisms by which GGT affects tumor biology have been investigated. As a membrane-bound enzyme, GGT plays an essential role in maintaining the production of intracellular glutathione (GSH), which protects cells from reactive oxygen compounds and free radicals as a key antioxidant element [45]. Therefore, GGT may contribute to the formation of tumor microenvironment that protects tumor cells form oxidative stress and drug cytotoxic effects [46,47]. Furthermore, reactive oxygen species (ROS), as a kind of carcinogenic factor, could up-regulate GGT expression through the redox regulation of many genes [5]. Therefore, it could be postulated that oxidative stress, as a part of the tumor microenvironment, might promote tumor tissues to produce GGT, and an elevated GGT expression might protect cells from the impacts of oxidative stress. For instance, up-regulation of GGT could help prostate epithelial cell overcome hydrogen peroxide-induced apoptosis [48], but this should be further studied in tumor cells. Additionally, GGT could also induce the production of additional source of endogenous ROS, leaving cells exposed to persistent oxidative stress, subsequently inducing aberrant CpG island methylation, DNA damage and genome instability, and ultimately promoting several carcinogenic processes, such as cellular growth, proliferation, and survival [5,7,49,50]. Evidences showed that several inflammatory cytokines, including tumor necrosis factor α, interferon-α, and interferon-β, could induce GGT expression [45,46]. Additionally, it was also reported that serum GGT level associates with the active status, fibrosis and cirrhosis stage of chronic hepatitis [51,52] and functions as a biomarker of the inflamed liver microenvironment in hepatitis [53]. Therefore, it may be hypothesized that GGT reflects or participates in the tumor-associated inflammatory responses to predict the prognosis of tumor patients. Abnormal Ras signaling transduction plays a key role in promoting cancer progression and closely correlates with the prognosis of cancer patients [54], and oxidative stress-induced activation of Ras-mitogen-activated protein kinase pathways could up-regulate GGT expression in colon cancer cells [55,56]. Thus, it is possible that elevated GGT reflects the abnormal activation of Ras signaling pathway to associate with the prognosis of tumor patients. However, the exact direct mechanisms of elevated GGT in cancer initiation and progression was rarely declared, so more studies should be performed in this regard.

A larger sample size was one of the strengths of our meta-analysis, which made our study have more statistical power than any of the included studies. In addition, our meta-analysis was performed by analyzing a massive dataset from multicenters and this made our conclusions more generalizable. However, there were also several limitations in our meta-analysis. First, most important limitation was that there was significant heterogeneity for data synthesis of OS. Although subgroup and sensitivity analyses were performed, the main source of heterogeneity was not identified. It may be possible that the significant heterogeneity derived from the inconsistencies in patient characteristics and study designs, and thus more homogeneous studies are required to validate our findings. Second, our study was a literature-based analysis, and thereby had a risk of publication bias, in which predominantly positive results had a tendency to be published, ultimately exaggerating our estimation for the relationship between serum GGT and survival. Third, the cut-off values applied to define the elevated GGT level across the included studies were inconsistent. For one thing, it might introduce the heterogeneity into our meta-analysis and weaken the reliability of our conclusions. For another thing, the different cut-off values made it difficult for doctors to make clinical decisions based on GGT level of patients with PLC. Finally, sometimes, serum GGT could have been influenced by other non-neoplastic conditions, such as the diseases of hepatobiliary tract, pancreatic, heart disease, and alcohol abuse. Moreover, some of the included studies in this meta-analysis did not clearly state that PLC patients with those conditions were excluded or explore the effects of those factors on the prognosis. Last but not the least, many cancer prognosis-associated variables, such as tumor grade, tumor differentiation, and additional treatment, were not available for sensitivity and subgroup analyses, since most of included studies did not provide enough information about these variables. Therefore, the pooled results in this meta-analysis may face unavoidable bias risks.

In conclusion, this meta-analysis demonstrated that higher serum GGT level was associated with poorer prognosis for PLC patients. The serum GGT was an economical and effective prognostic biomarker, which could be applied for risk stratification and formulating individualized treatments for PLC patients. Considering the limitation of our meta-analysis analysis, more prospectively well-designed studies are demanded to confirm our findings and meanwhile investigate the correspondent mechanisms deeply.

## Supporting information

**supplementary Material:** 

## Abbreviations

CI: confidence interval
DFS: disease-free survival
HCC: hepatocellular carcinoma
HR: hazard ratio
ICC: intrahepatic cholangiocarcinoma
NOS: Newcastle–Ottawa scale
OS: overall survival
PLC: primary liver cancer
RFS: recurrence-free survival
ROS: reactive oxygen species
TACE: transcatheter arterial chemoembolization
