# Supplementary material for: The prognostic significance of pretreatment serum γ-glutamyltranspeptidase in primary liver cancer: a meta-analysis and systematic review
Source: Biosci Rep. 2018 Nov 28;38(6):BSR20181058. doi: 10.1042/BSR20181058 (PMC6259011; doi:10.1042/BSR20181058)
Supplement: Supplementary file 1 [file bsr20181058_Supp1.pdf]

1. Detailed search strategy in PubMed

((((((((hepatocellular carcinoma[Title/Abstract]) OR intrahepatic cholangiocarcinoma[Title/Abstract]) OR liver cancer[Title/Abstract]) OR liver primary cancer[Title/Abstract]) OR liver primary carcinoma[Title/Abstract]) OR liver carcinoma[Title/Abstract])) AND ((((survival[Title/Abstract]) OR prognosis[Title/Abstract]) OR prognostic[Title/Abstract]) OR outcome[Title/Abstract])) AND ((((((((Gamma-glutamyltranspeptidase[Title/Abstract]) OR Gamma-glutamyl transpeptidase[Title/Abstract]) OR γ-glutamyltranspeptidase[Title/Abstract]) OR γ-glutamyl transpeptidase[Title/Abstract]) OR Gamma-glutamyltransferase[Title/Abstract]) OR Gamma-glutamyl transferase[Title/Abstract]) OR γ-glutamyltransferase[Title/Abstract]) OR γ-glutamyl transferase[Title/Abstract])

2. Detailed search strategy in Web of Science

#1 TS= ("hepatocellular carcinoma" OR "intrahepatic cholangiocarcinoma" OR" liver cancer" OR "liver primary cancer" OR "liver primary carcinoma" OR "liver carcinoma")

#2 TS= (survival OR prognosis OR prognostic OR outcome)

#3 TS= ("Gamma-glutamyltranspeptidase" OR "Gamma-glutamyl transpeptidase" OR "γ-glutamyltranspeptidase" OR "γ-glutamyl transpeptidase" OR "Gamma-glutamyltransferase" OR "Gamma-glutamyl transferase" OR "γ-glutamyltransferase" OR "γ-glutamy ltransferase")

#4 #3 AND #2 AND #1

3. Detailed search strategy in EMBASE

#1 'hepatocellular carcinoma':ab,ti
#2 'intrahepatic cholangiocarcinoma':ab,ti
#3 'liver primary cancer':ab,ti
#4 'liver primary carcinoma':ab,ti
#5 'liver cancer':ab,ti
#6 'liver carcinoma':ab,ti
#7 #1 OR #2 OR #3 OR #4 OR #5 OR #6
#8 'survival':ab,ti
#9 'prognosis':ab,ti
#10 'prognostic':ab,ti
#11 'outcome':ab,ti
#12 #8 OR #9 OR #10 OR #11
#13 'gamma-glutamyl transpeptidase':ab,ti
#14 'gamma-glutamyltranspeptidase':ab,ti
#15 'γ-glutamyltranspeptidase':ab,ti

#16 'γ-glutamyl transpeptidase':ab,ti
#17 'gamma-glutamyltransferase':ab,ti
#18 'gamma-glutamyl transferase':ab,ti
#19 'γ-glutamyltransferase':ab,ti
#20 'γ-glutamyl transferase':ab,ti
#21 #13 OR #14 OR #15 OR #16 OR #17 OR #18 OR #19 OR #20
#22 #7 AND #12 AND #21
